# Impact of Excipient and Cell Concentration on the Viability, Proliferation, and Adhesion of Mesenchymal Stem Cells: Future Relevance for the Development of a New Advanced Therapy Medicinal Product

**DOI:** 10.3390/pharmaceutics17050642

**Published:** 2025-05-13

**Authors:** Ester Moñivas, Concepción Aguayo, Beatriz Rodera, Mercedes Zurita

**Affiliations:** 1 Fundación Investigación Biomédica, Hospital Universitario Puerta de Hierro Majadahonda, 28222 Majadahonda, Spain; 2 Hospital Universitario Puerta de Hierro Majadahonda, 28222 Majadahonda, Spain; conchiaguaferr@hotmail.com (C.A.); brodera23@gmail.com (B.R.);

**Keywords:** mesenchymal stem cells, advanced therapy medicinal products, human platelet lysate, Hypothermosol

## Abstract

**Introduction**: The preservation of mesenchymal stem cell (MSC) viability and biological activity is a key aspect in optimizing advanced therapy medicinal products (ATMPs). Evaluating various excipients to optimize MSC conservation and functionality is essential. **Methods**: Five excipients with different proportions of human platelet lysate (hPL) and Hypothermosol were evaluated at two different cell concentrations (0.1 × 10^6^ MSC/μL and 0.008 × 10^6^ MSC/μL). Cell viability, adhesion, and proliferation capacity were assessed at 24 and 48 h under hypothermic conditions (2–8 °C). **Results**: A significant interaction was observed between cell concentration and excipient, where the 0.008 × 10^6^ MSC/μL concentration showed better viability results. Excipients with a combination of 50–75% Hypothermosol improved cell viability and adhesion. No significant differences were found in cell proliferation among the excipients studied. Viability, adhesion, and proliferation decreased significantly at 48 h for all excipients and concentrations evaluated. **Conclusions**: The combination of hPL and Hypothermosol enhances MSC stability and preserves their functionality, suggesting its potential as an optimized storage solution for cell-based therapies. Additionally, the impact of cell concentration on viability underscores the importance of selecting appropriate dosing. Future studies should further investigate how these findings translate into clinical outcomes, particularly in terms of therapeutic efficacy and patient safety.

## 1. Introduction

In recent years, the rise of advanced therapies has revolutionized the treatment of numerous pathologies that previously lacked effective treatments. Advanced therapies primarily encompass innovative treatments involving genes, tissues, or cells, tailored to patient needs in what is known as personalized medicine. Advanced Therapy medicinal products (ATMPs) include autologous, allogeneic, or xenogeneic products. Among the most promising ATMPs are cell-based therapies. However, these therapies face challenges, particularly regarding cell viability preservation during handling and storage, as well as compliance with complex regulatory standards to ensure patient safety and efficacy [1,2].

Mesenchymal stem cells (MSCs) are multipotent stem cells that adhere to culture surfaces, exhibit a fibroblast-like morphology, and can differentiate into mesodermal-derived cells such as osteocytes, chondrocytes, and adipocytes. This makes them a promising option for therapeutic applications in advanced therapy medicinal products. MSCs can be obtained from various sources, including adipose tissue, umbilical cord, and bone marrow [3]. Bone marrow has been established as the most commonly used source due to its accessibility and abundance [4,5]. In addition to their differentiation capacity, MSCs also exhibit immunomodulatory and anti-inflammatory properties, making them ideal candidates for treating various diseases and disorders [6].

Since MSCs are living cells requiring strictly controlled conditions to maintain their properties, viability, and functional stability, selecting the appropriate excipient is a crucial factor. In this context, human platelet lysate (hPL) has emerged as an effective alternative to animal-derived sera, providing a medium rich in growth factors and bioactive cytokines that support MSC growth and functionality. hPL reduces the risk of immune reactions and contamination associated with animal sera, thereby improving the safety and efficacy of cell-based medicinal products [7,8]. Similarly, Hypothermosol, a specialized cell preservation solution, has gained importance in the ATMP field due to its ability to optimize cell viability and functionality during low-temperature storage. Its isotonic formulation with pH buffers reduces thermal and osmotic stress on cells and tissues. Hypothermosol protects MSCs by reducing oxidative stress, stabilizing cell membranes, preserving structural integrity, and minimizing apoptosis [9,10].

This study aims to evaluate the impact of different combinations of human platelet lysate and Hypothermosol under hypothermic conditions, analyzing their effect on cell viability, proliferation, and functional stability over 48 h.

## 2. Materials and Methods

### 2.1. Sample Collection and Cell Culture

Mononuclear cells (MNCs) remaining from the bone marrow aspiration of patients scheduled for treatment at the Cellular Therapy Unit with the autologous advanced therapy medicinal product NC1, authorized under Authorization No. 83976 by the Spanish Agency for Medicines and Medical Devices (AEMPS), were used. Patient selection was conducted by a multidisciplinary committee at Puerta de Hierro University Hospital based on predefined clinical criteria to initiate the manufacturing process of the NC1 medicinal product. All patients were fully informed about the study and provided written informed consent.

MNCs were initially cultured in minimum essential medium (α-MEM; Bio-Whittaker, Lonza Group, Basel, Switzerland) supplemented with 5% hPL (Bexen, Hernani, Spain), 10 mmol glutamine (GibcoBRL, Thermo Fisher Scientific, Waltham, MA, USA), and 1X antibiotic–antimycotic solution (GibcoBRL, Thermo Fisher Scientific, Waltham, MA, USA, diluted 1:100 from 100X stock) to allow the selection of MSCs. Since MSCs are the only cell type within the MNC fraction capable of adhering to plastic, non-adherent cells were progressively removed through medium changes. Once MSC cultures reached ≥90% confluence, cell formulation was carried out for the different study groups.

### 2.2. Study Group Formulation

Two different MSC concentrations were evaluated. The first concentration studied was 0.1 × 10^6^ MSC/μL, and the second was 0.008 × 10^6^ MSC/μL. For each of these concentrations, five experimental groups were established based on the combination of excipients used. The first group consisted of the active substance (MSC) combined with an excipient composed of 100% hPL. The second group included the active substance in an excipient mixture composed of 75% hPL and 25% Hypothermosol. In the third group, the active substance was combined with an excipient composed of 50% hPL and 50% Hypothermosol. The fourth excipient consisted of a mixture of 25% hPL and 75% Hypothermosol along with the active substance. Finally, the fifth group included the active substance with an excipient composed solely of 100% Hypothermosol.

### 2.3. Cell Viability Assessment at Study Time Points

The viability of the formulated product stored at 2–8 °C was evaluated at 24 and 48 h. Cell counts were performed at these time points using the standard Trypan Blue dye exclusion method.

### 2.4. Cell Proliferation Assessment—Ki67 Immunohistochemistry

Mesenchymal stem cells were seeded at a concentration of 2000/cm^2^ onto culture slides and incubated at 37 °C in a 5% CO_2_ atmosphere. After 24 h, the slides were fixed in 4% paraformaldehyde for 30 min and washed with distilled water for 10 min, followed by three 5 min washes with PBS 1X (Thermo Fisher Scientific, Waltham, MA, USA). Antigen retrieval was performed by treating the sections with citrate buffer (pH 6.0) for 20 min in a heat cooker, followed by three washes with PBS 1X. Next, the slides were incubated with 30% hydrogen peroxide to block endogenous peroxidase activity and washed with PBS 1X.

The slides were then incubated for 1 h at room temperature with a non-immune serum blocking solution to prevent nonspecific binding. Subsequently, the primary Rabbit Anti-Human Ki-67 Monoclonal Antibody (Master Diagnostica, Madrid, Spain) was added and incubated overnight at 4 °C in a humid chamber. After primary antibody incubation, slides were washed with PBS 1X, and a biotinylated secondary antibody (Master Polymer Plus HRP, Granada, Spain) at a 1/200 dilution in blocking solution was added, followed by a 1 h incubation at room temperature in a humid chamber. The secondary antibody was revealed using an ABC complex (streptavidin) incubated for 1 h at room temperature in a humid chamber.

To develop the color reaction, a diaminobenzidine chromogen (Master Diagnostic Developing Kit, Madrid, Spain) was applied for a maximum of 15 min at room temperature. Contrast staining was performed with hematoxylin for 3 min, and the slides were mounted with EUKIT. Marker expression was evaluated using the Ki67 labeling index, expressed as a percentage. The stained cells were counted relative to the total cell number.

### 2.5. Cell Adhesion Assessment

To obtain adherent cells, MSCs were seeded at a concentration of 10,000 cells/cm^2^ in 175 cm^2^ culture flasks (Thermo Fisher Scientific, Waltham, MA, USA) using α-MEM (BioWhittaker, Lonza Group, Basel, Switzerland) supplemented with 5% hPL (Bexen, Hernani, Spain), 10 mmol glutamine (GibcoBRL, Thermo Fisher Scientific, Waltham, MA, USA), and 1X antibiotic–antimycotic solution (GibcoBRL, diluted 1:100 from 100X stock). The cells were incubated at 37 °C in a fully humidified atmosphere with 5% CO_2_. After 24 h, adherent cells were washed twice with PBS 1X and detached using a trypsin/ethylenediaminetetraacetic acid (EDTA) solution (BioWhittaker, Lonza Group, Basel, Switzerland) for 15 min at 37 °C.

Trypsin neutralization and subsequent washing were performed using α-MEM (BioWhittaker, Lonza Group, Basel, Switzerland) supplemented with 5% hPL (Bexen, Hernani, Spain), 10 mmol glutamine (GibcoBRL, Thermo Fisher Scientific, Waltham, MA, USA), and 1X antibiotic–antimycotic solution (GibcoBRL, Thermo Fisher Scientific, Waltham, MA, USA, diluted 1:100 from 100X stock). Adherent MSCs were centrifuged at 1250 rpm for 10 min and resuspended in α-MEM supplemented with 5% hPL (Bexen, Hernani, Spain) and 10 mmol glutamine (GibcoBRL, Thermo Fisher Scientific, Waltham, MA, USA). Adherent cell counts were performed using the standard Trypan Blue exclusion method.

### 2.6. Statistical Analysis

A mixed-effect linear regression model was used for data analysis. This statistical method allows for handling data by adjusting both fixed and random effects. Fixed effects included variables such as different excipient groups, cell concentration, and time, while random effects accounted for intra-group variability.

This approach was used to evaluate cell viability and other variables of interest over time and under different experimental conditions. The model also considered residuals to verify model fit and ensure compliance with analysis assumptions, such as normal error distribution and homoscedasticity.

## 3. Results

### 3.1. Cell Viability

The initial mean cell viability was 93.51 ± 4.52 for all study groups. A multilevel mixed linear regression analysis was performed to evaluate cell viability as a function of the excipient, the concentration used, and time (24 and 48 h). Time had a significant impact on viability, with an average reduction of 4.03 units at 48 h compared to 24 h (*p* < 0.001).

**Figure 1:**
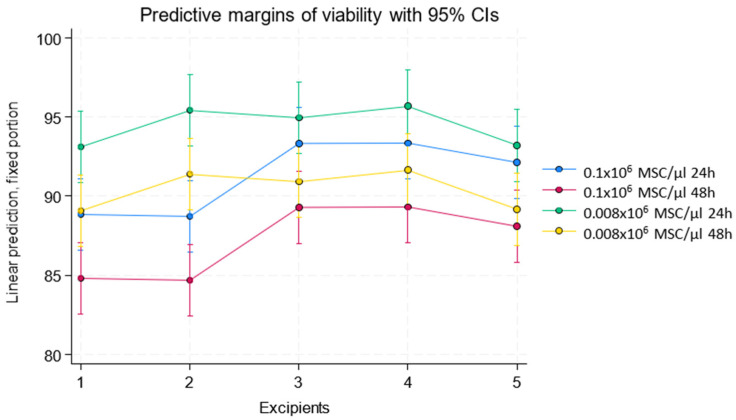
Viability of MSCs after 24 and 48 h in five different excipients (100% hPL (1), 75% hPL and 25% Hypothermosol (2), 50% hPL and 50% Hypothermosol (3), 25% hPL and 75% Hypothermosol (4), and 100% Hypothermosol (5)) and two cell concentrations (0.1 × 10^6^ and 0.008 × 10^6^ MSC/μL). Higher viability was observed in formulations containing ≥50% Hypothermosol (excipients 3, 4, and 5). A significant decrease is observed at 48 h across all groups.

A significant interaction was found between the excipient and the cell concentration used (0.1 × 10^6^ MSC/μL and 0.008 × 10^6^ MSC/μL). Linear predictions for viability showed that, with excipient 1, samples achieved an average viability of 86.82 units with the 0.1 × 10^6^ MSC/μL concentration and 91.09 with the 0.008 × 10^6^ MSC/μL concentration. Excipient 2 showed 86.70 units with the 0.1 × 10^6^ MSC/μL concentration and 93.41 with the 0.008 × 10^6^ MSC/μL concentration. Excipient 3 showed 91.30 units with the 0.1 × 10^6^ MSC/μL concentration and 92.94 with the 0.008 × 10^6^ MSC/μL concentration. Excipient 4 showed 91.33 units with the 0.1 × 10^6^ MSC/μL concentration and 93.67 with the 0.008 × 10^6^ MSC/μL concentration, while excipient 5 showed 90.10 units with the 0.1 × 10^6^ MSC/μL concentration and 91.18 with the 0.008 × 10^6^ MSC/μL concentration (results shown in Table 1 and Figure 1).

**Figure 2:**
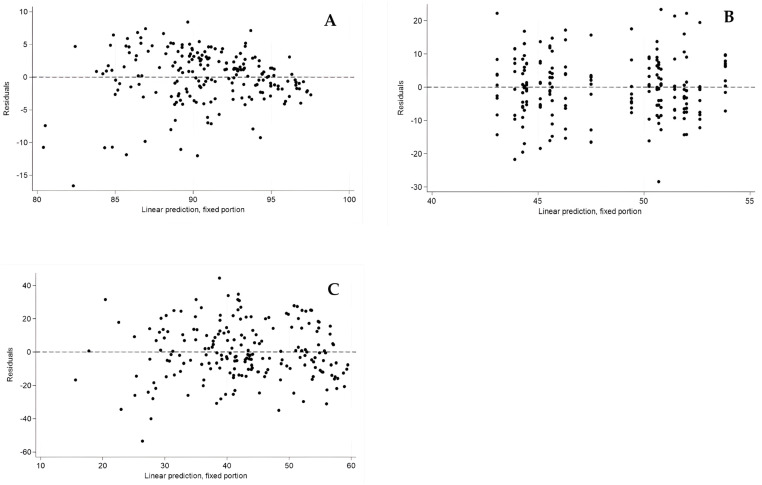
Evaluation of the mixed-effect linear regression model assumptions applied to data on cell viability (**A**), Ki67 expression as a proliferation marker (**B**), and cell adhesion (**C**). The residuals are randomly distributed around zero, with no clear patterns or signs of heteroscedasticity.

No differences were found between excipients 1 and 2. Excipients 3, 4, and 5 showed higher viability compared to excipients 1 and 2. On average, excipient 3 had 4.48 more viability units than excipient 1 (*p* = 0.001), excipient 4 had 4.50 more units (*p* = 0.001), and excipient 5 had 3.28 more units (*p* = 0.012). These results are supported by the residual analysis, which confirmed the homoscedasticity assumption. The standardized residuals for the cell viability model showed no discernible trends and were randomly distributed around zero, validating the adequacy of the fitted model (Figure 2A).

### 3.2. Cell Adhesion

**Figure 3:**
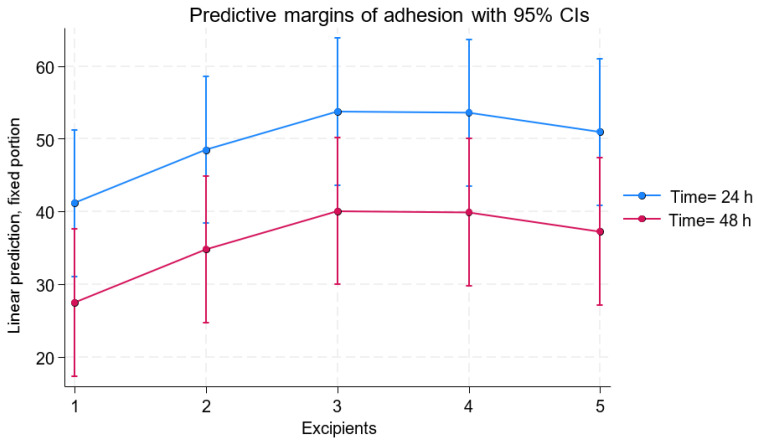
Adhesion of MSCs after 24 and 48 h in five different excipients (100% hPL (1), 75% hPL and 25% Hypothermosol (2), 50% hPL and 50% Hypothermosol (3), 25% hPL and 75% Hypothermosol (4), and 100% Hypothermosol (5)). Excipients containing 50–75% Hypothermosol (excipients 3 and 4) promote greater adhesion. A significant decrease is observed at 48 h across all groups.

**Figure 4:**
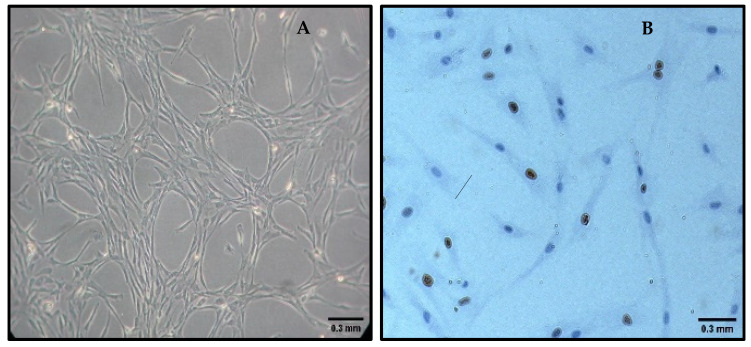
Evaluation of adherence and proliferation in MSC cultures. Representative image of adherent MSCs in culture (**A**). Immunophenotypic identification of the proliferation marker Ki67. The black arrow indicates a Ki67-positive cell, highlighted by nuclear staining (**B**).

A multilevel mixed linear regression analysis was performed to evaluate cell adhesion as a function of the excipient, the concentration used, and time (24 and 48 h) (Figure 3 and Figure 4A). No statistically significant differences were found in the evaluation of the concentration used (*p* = 0.965). The differences observed among the excipients showed that excipient 2 increased cell adhesion by 6.35 units compared to excipient 1 (*p* = 0.049). Excipient 3 showed a significant increase of 10.09 adhesion units compared to excipient 1 (*p* = 0.002), followed by excipient 4 with 9.74 more units (*p* = 0.003) and excipient 5 with 8.13 more adhesion units (*p* = 0.012).

Evaluation time was an important factor, as a significant decrease in actual viability was detected at 48 h, with 10.46 fewer units compared to 24 h (*p* < 0.001) (results shown in Figure 2 and Table 2). Additionally, residual analysis indicated a random distribution without signs of heteroscedasticity, supporting the appropriateness of the regression model used to assess cell adhesion (Figure 2C).

### 3.3. Ki67 Marker Expression

A multilevel mixed linear regression analysis was performed to evaluate Ki67 expression as a function of the excipient, the concentration used, and time (24 and 48 h). No statistically significant differences were found in Ki67 expression among the excipients (Figure 4B). Compared to excipient 1, excipient 2 showed a difference of −3.22 units (*p* = 0.125). Excipient 3 showed a difference of −1.83 units (*p* = 0.384), followed by excipient 4 with a difference of −2.39 units (*p* = 0.255) and excipient 5 with a difference of −1.94 units (*p* = 0.350). Similarly, cell concentration had no significant effect on Ki67 expression (*p* = 0.370). Evaluation time showed a significant decrease in Ki67 expression at 48 h compared to 24 h (coefficient = −6.34, *p* < 0.001). Residual analysis showed no evidence of heteroscedasticity, with standardized residuals evenly dispersed around zero, supporting the validity of the model applied to Ki67 expression data (Figure 2B).

## 4. Discussion

In recent decades, there has been an exponential increase in advanced therapy medicinal products, including cell-based therapies classified as cellular therapy. This increase is reflected in the growing number of clinical trials registered on the clinicaltrials.gov website, with a total of 1731 trials involving mesenchymal stem cell-based therapies. One of the most significant limitations of these therapies is the short shelf life of these medicinal products, due to the specific in vitro culture and handling requirements of the cells and the need to develop excipients capable of maintaining proper physiological functions and long-term cell stability. This limitation highlights the need to optimize cell storage conditions by developing new excipients that ensure proper preservation and storage, maintaining cell viability and therapeutic potential while achieving a consistent and extended shelf life. Most commercially available cell stabilization fluids are not suitable for direct patient infusion [10]. Due to this limitation, the use of human platelet lysate, Hypothermosol—designed for cell maintenance and preservation without the need for removal before patient administration—and a combination of both substances in different proportions has been proposed as excipients capable of maintaining the viability, proliferation, and adhesion of mesenchymal stem cells under hypothermic conditions (2–8 °C) over extended periods, with studies conducted at 24 and 48 h post-formulation at two different concentrations, 0.1 × 10^6^ MSC/μL and 0.008 × 10^6^ MSC/μL.

In recent years, a growing number of publications have focused on the use of hPL for MSC storage. Recent studies have demonstrated that hPL can enhance the post-thaw recovery and functionality of MSCs, whether used alone or in combination with other excipients [11,12]. In our study, hPL was evaluated as a sole component of the excipient, as well as in combination with Hypothermosol. Our findings indicate that cell cultures with higher initial viability tended to maintain higher viability levels in studies conducted at 24 and 48 h. Additionally, it was found that the combination of platelet lysate and Hypothermosol, in proportions of 50–75% Hypothermosol, resulted in the highest viability outcomes in both 0.1 × 10^6^ MSC/μL and 0.008 × 10^6^ MSC/μL concentrations. However, an interaction between excipients and cell concentration was observed, with significant differences in viability results. It was found that the 0.008 × 10^6^ MSC/μL concentration exhibited higher cell viability than the 0.1 × 10^6^ MSC/μL concentration, suggesting that a higher cell density could induce stress or affect nutrient availability. These results highlight that although human platelet lysate contains numerous factors such as cytokines, chemokines, adhesion factors, and various growth factors that modulate tissue repair and cell growth—including PDGF-AA, PDGF-AB/BB, transforming growth factor-β1 (TGF-β1), epidermal growth factor, basic fibroblast growth factor (bFGF), insulin-like growth factor 1, and vascular endothelial growth factor [13,14,15]—a higher cell concentration may reduce the availability of these factors. Additionally, the presence of Hypothermosol enhances MSC stability and viability, likely due to its antioxidant and stabilizing properties, which reduce oxidative stress and maintain cellular integrity during storage. When evaluating cell viability over time, results showed an average viability loss of 4.03 units at 48 h compared to 24 h, regardless of the excipient studied, indicating a predictable decline in cell integrity over time. These findings underscore the importance of combining human platelet lysate, as a source of cytokines, adhesion factors, and growth factors, with Hypothermosol as a stabilizing and protective solution for cells under hypothermic conditions. This combination increases shelf life, as cell viability is a critical parameter in ensuring the therapeutic efficacy of ATMPs [9,10,16,17,18,19,20].

The results of the cell proliferation analysis, based on Ki67 marker expression, showed no statistically significant differences among the different excipients or cell concentrations tested. This methodology was selected due to the established role of Ki67 as a reliable marker of proliferative activity in cultured MSCs. While reference [21] supports the suitability of Ki67 as a methodological tool, our interpretation of the observed outcomes is grounded solely in the data obtained from this study. The absence of differences suggests that MSC proliferation was not markedly influenced by the tested excipients or concentrations under the experimental conditions used. Although no differences in cell proliferation were observed, time was found to have a significant impact on MSC proliferative capacity, with a reduction of 6.34 activity units, suggesting a potential transition of the cells to a lower metabolic activity state over time.

The study of cell adhesion did not show significant differences between the two concentrations tested, but a significant improvement in cell adhesion capacity was found with excipients containing a combination of platelet lysate and Hypothermosol in proportions of 50–75% Hypothermosol—the same excipients that showed the highest viability results in both 0.1 × 10^6^ MSC/μL and 0.008 × 10^6^ MSC/μL concentrations. The adhesion factors present in human platelet lysate [13,14,15], combined with the protective function of Hypothermosol, may promote MSC adhesion to culture surfaces [10].

### Limitations and Future Directions

Despite these promising results, several limitations of the present study should be acknowledged. First, the use of the Trypan Blue exclusion method, although widely employed due to its simplicity and cost-effectiveness, has inherent limitations in sensitivity. This technique does not discriminate between cells in early apoptotic stages and truly viable cells, potentially leading to an overestimation of viability. Moreover, recent findings have shown that Trypan Blue can induce the rupture of dead or dying cells, further compromising the accuracy of cell counts and potentially underestimating the proportion of non-viable cells [22]. Furthermore, emerging storage solutions, such as PEG-polyalanine thermogel, which has been reported to achieve up to 2.5-fold higher cell recovery compared to HTS, deserve consideration, although their use remains investigational, and they have yet to obtain regulatory approval [23].

Overall, these results emphasize the importance of selecting excipients that not only preserve cell viability but also maintain MSC functionality. The combination of human platelet lysate and Hypothermosol emerges as a promising strategy for improving the stability and efficacy of ATMPs. These findings lay the groundwork for future studies focused on clinical applications, aiming to maximize the viability and functionality of stem cells in advanced therapy medicinal products.

## Figures and Tables

**Table 1:** Summary of cell viability results by excipient and concentration.

Excipient	Concentration	Mean Viability (24 h)	[95% Conf. Interval]	Mean Viability (48 h)	[95% Conf. Interval]	Change over Time	*p*-Value
**100% HPL**	0.1 × 10^6^ MSC/μL	88.84 ± 1.16	[86.57–91.11]	84.80 ± 1.16	[82.53–87.04]	−2.10	<0.001
	0.008 × 10^6^ MSC/μL	93.11 ± 1.16	[90.84–95.38]	89.07 ± 1.16	[86.80–91.34]	−3.07	<0.001
**25% HPL/75% HYP**	0.1 × 10^6^ MSC/μL	88.72 ± 1.16	[86.45–90.99]	84.68 ± 1.16	[82.41–86.96]	−2.14	<0.001
	0.008 × 10^6^ MSC/μL	95.43 ± 1.16	[93.16–97.70]	91.39 ± 1.16	[89.12–93.67]	−3.14	<0.001
**50% HPL/50% HYP**	0.1 × 10^6^ MSC/μL	93.32 ± 1.16	[91.05–95.59]	89.28 ± 1.16	[87.01–91.56]	−2.85	<0.001
	0.008 × 10^6^ MSC/μL	94.96 ± 1.16	[92.69–97.23]	90.92 ± 1.16	[88.65–93.20]	−3.27	<0.001
**75% H** **PL** **/25% HYP**	0.1 × 10^6^ MSC/μL	93.34 ± 1.16	[91.07–95.62]	89.31 ± 1.16	[87.04–91.58]	−2.83	<0.001
	0.008 × 10^6^ MSC/μL	95.69 ± 1.16	[93.42–97.96]	91.65 ± 1.16	[89.38–93.92]	−3.32	<0.001
**100% HYP**	0.1 × 10^6^ MSC/μL	92.12 ± 1.16	[89.85–94.39]	88.08 ± 1.16	[85.81–90.36]	−3.33	<0.001
	0.008 × 10^6^ MSC/μL	93.21 ± 1.16	[90.93–95.48]	89.17 ± 1.16	[86.90–91.44]	−3.36	<0.001

**Table 2:** Loss of cell adhesion from 24 to 48 h.

Excipient	Adhesion at 24 h (Mean ± SD)	[95% Conf. Interval]	Adhesion at 48 h (Mean ± SD)	[95% Conf. Interval]	*p*-Value
**100% HPL**	41.15 ± 5.15	[31.04–51.24]	27.44 ± 5.15	[17.34–37.55]	<0.001
**25% HPL/75% HYP**	48.48 ± 5.15	[38.38–58.58]	34.78 ± 5.15	[24.68–44.88]	<0.001
**50% HPL/50% HYP**	53.75 ± 5.15	[43.65–63.86]	40.06 ± 5.15	[29.95–50,16]	<0.001
**75% HPL/25% HYP**	53.59 ± 5.15	[43.49–63.70]	39.90 ± 5.15	[29.79–50.00]	<0.001
**100% HYP**	50.93 ± 5.15	[40.83–61.04]	37.24 ± 5.15	[27.13–47.34]	<0.001

## Data Availability

The original contributions presented in this study are included in the article. Further inquiries can be directed to the corresponding author.
